# Condensation patterns of prophase/prometaphase chromosome are correlated with H4K5 histone acetylation and genomic DNA contents in plants

**DOI:** 10.1371/journal.pone.0183341

**Published:** 2017-08-30

**Authors:** Lidiane Feitoza, Lucas Costa, Marcelo Guerra

**Affiliations:** Laboratory of Plant Cytogenetics and Evolution, Department of Botany, Federal University of Pernambuco, Recife, PE, Brazil; Virginia Tech, UNITED STATES

## Abstract

Mitotic prophase chromosome condensation plays an essential role in nuclear division being therefore regulated by highly conserved mechanisms. However, degrees of chromatin condensation in prophase-prometaphase cells may vary along the chromosomes resulting in specific condensation patterns. We examined different condensation patterns (CPs) of prophase and prometaphase chromosomes and investigated their relationship with genome size and distribution of histone H4 acetylated at lysine 5 (H4K5ac) in 17 plant species. Our results showed that most species with small genomes (2C < 5 pg) (*Arachis pusilla*, *Bixa orellana*, *Costus spiralis*, *Eleutherine bulbosa*, *Indigofera campestris*, *Phaseolus lunatus*, *P*. *vulgaris*, *Poncirus trifoliata*, and *Solanum lycopersicum*) displayed prophase chromosomes with late condensing terminal regions that were highly enriched in H4K5ac, and early condensing regions with apparently non-acetylated proximal chromatin. The species with large genomes (*Allium cepa*, *Callisia repens*, *Araucaria angustifolia* and *Nothoscordum pulchellum*) displayed uniformly condensed and acetylated prophase/prometaphase chromosomes. Three species with small genomes (*Eleocharis geniculata*, *Rhynchospora pubera*, and *R*. *tenuis*) displayed CP and H4K5ac labeling patterns similar to species with large genomes, whereas a forth species (*Emilia sonchifolia*) exhibited a gradual chromosome labeling, being more acetylated in the terminal regions and less acetylated in the proximal ones. The nucleolus organizer chromatin was the only chromosomal region that in prometaphase or metaphase could be hyperacetylated, hypoacetylated or non-acetylated, depending on the species. Our data indicate that the CP of a plant chromosome complement is influenced but not exclusively determined by nuclear and chromosomal DNA contents, whereas the CP of individual chromosomes is clearly correlated with H4K5ac distribution.

## Introduction

Chromatin condensation is a major step in the cell cycle, enabling the spatial separation of each chromosome, their mobility to the equatorial plane, and correct segregation during anaphase. The processes involved in chromosome condensation are largely promoted by protein complexes called condensins, but also involve histone modifications and the combined actions of several other nuclear proteins and non-coding RNAs [1–2]. The condensation timing of each chromosome region is inversely correlated with its replication time: late replicating chromatin (including heterochromatin) is early condensing, whereas early replicating chromatin (gene-rich euchromatin) is late condensing [3–4]. As a consequence, prophase chromosomes often appear differentiated into heteropycnotic regions (precociously condensed, and deeply stained chromatin) and eupycnotic regions (under-condensed and less stained chromatin), using classical cytological staining techniques [5]. The resulting condensation patterns (CPs) are quite variable among species, being responsible for the longitudinal differentiation observed, for example, in the pachytene chromosomes of many plant and animal species or in the G (more condensed) and R (less condensed) euchromatic bands of vertebrate chromosomes [1].

The mitotic prophase-prometaphase chromosomes of plants may show different CPs, characterized by varying proportions of early and late condensing chromatin. The different proportions of early and late condensing chromatin may be enough to characterize each chromosome of a karyotype [6–8]. Species with small chromosomes, such as *Arabidopsis thaliana* and *Theobroma cacao*, typically show prophase chromosomes clearly differentiated into early-condensed proximal chromatin and late-condensed terminal chromatin [9–10]. A quite distinct CP is observed in species with large chromosomes, such as *Allium cepa* and *Vicia faba*, which have uniformly condensed prophase chromosomes [11]. These two extreme patterns will be referred to here as the Arabidopsis-type and the Allium-type respectively. Between these extremes, many intermediate CPs have been observed, displaying variable proportions of early and late condensing chromatin, as for example among species of *Solanum* or orchids [12, 13]. The intermediate patterns can be grouped into Solanum-type (chromosomes generally small, more similar to the Arabidopsis-type but with a higher proportion of proximal condensed chromatin) and Hordeum-type (medium sized chromosomes similar to Allium-type but less uniformly condensed during prophase). In some cases, however, the characterization of prophase chromosomes as belonging to Arabidopsis- or Solanum-type or to Hordeum- or Allium-type is not easy. These four types of prophase chromosomes were originally described as Cucurbita-, Fatshedera-, Pinus- and Allium-type [14]. The three first genera were changed here by more widely known genera, having in mind *A*. *thaliana*, *S*. *lycopersicum*, and *H*. *vulgare* as respective models.

CPs are strongly associated with the chromatin organization observed in interphase [11, 14], which has been formerly more intensively investigated (reviewed by Delay, [15]). Species with the Arabidopsis-type CP display interphase nuclei with well-defined chromocenters immersed in diffuse and weakly stained euchromatin. This type of nucleus is denominated *areticulate* because its chromatin reticulum is almost invisible, especially after Feulgen staining. Differently, species with *Allium*-type CP typically display nuclei with dense and uniformly stained chromatin, referred to as *eureticulate* nuclei. Nuclei of species with the Solanum-type CP show irregularly condensed chromatin and poorly defined chromocenters (*semi-reticulate* nuclei), whereas those of Hordeum-type are similar to Allium-type but exhibit a less dense and less regularly distributed chromatin (*reticulate nuclei*).

The different structural types of interphase nuclei and CPs are more or less correlated with nuclear DNA amount, mean DNA content per chromosome, and chromosome size. In general, Arabidopsis- and Solanum-type CPs are found in species with low nuclear DNA content, whereas Hordeum- and Allium-types are usually found in species with higher DNA content [16, 17]. Similarly, a correlation between genome size and chromosomal distribution of some posttranslationally modified histones has been reported for plant species. For example, the chromosomal distribution of the histone H3 dimethylated at lysine 4 (H3K4me2) in *Arabidopsis thaliana* (130 Mpb), *H*. *vulgare* (5440 Mpb), and *V*. *faba* (12810 Mpb), was distinct to each species, but it was almost identical among species with similar chromosome size and nuclear DNA content [18]. In *V*. *faba* and *H*. *vulgare*, both with large chromosomes, the labeling pattern observed with H3K4me2 was very similar to that obtained with the histone H4 acetylated at lysine 5 (H4K5ac) [19,20]. Likewise, in *Costus spiralis* and *Eleutherine bulbosa*, both with small chromosomes and Solanum-type CP, the distribution of H3K4me2 and H4K5ac signals were identical [21], suggesting that a general correlation between the distribution of these histones and CPs may exist. Actually, chromosome regions enriched in H3K4me2 and H4K5ac were found to be early replicating [18,22], therefore, they are expected to be late condensing.

H4K5ac is a universal mark for euchromatin and is directly involved in the control of chromatin replication and indirectly associated to chromatin condensation [23–25]. In general, early condensing chromatin (such as heterochromatin, G bands, and epignetically silenced chromatin, as the inactive X chromosome of female mammals) is hypoacetylated in H4K5 [25–27]. In the few plant species where the chromosomal distribution of H4K5ac was investigated by immunodetection, different patterns of acetylation were observed [21,22,28], but systematic efforts to correlate acetylation and CP have not been undertaken. It is interesting to note that the acetylation level of H4K5 in some chromosome sites may change during the cell cycle [20,23–24,29–30]. Further, specific chromatin types, as the nucleolus organizer region (NOR), may be hyperacetylated in some species [19,31] and hypoacetylated or non-acetylated in others [21], indicating that not all epigenetic marks are conserved in plants.

In this work, we analyzed the CPs, the nuclear and chromosomal DNA contents and the distribution of H4K5ac in 17 plant species with different chromosome sizes. To identify the acetylation levels of eu- or heterochromatic regions, the chromosomes were sequentially stained with the fluorochromes chromomycin A3 (CMA) and 4'-6-diamidino-2-phenylindole (DAPI) (which differentially stains most of the C-band heterochromatin) after immunodetection of H3K5ac. The distribution of heterochromatic bands was previously determined by C or CMA/DAPI banding for most of the species examined here (*e*.*g*., [12,32–37]). We have formerly reported that the hyperacetylated chromatin fraction observed in prophase chromosomes remains roughly the same in metaphase chromosomes [21]. Since the distribution of H4K5ac signals is more clearly defined when chromosomes are more condensed and separated, during prometaphase to metaphase, the characterization of the distinct acetylation patterns in the present sample was mainly performed in cells in these stages.

## Materials and methods

### Plant material

Seeds of *Araucaria angustifolia*, *Phaseolus vulgaris*, *and P*. *lunatus* and bulbs of *A*. *cepa* were obtained from commercial sources. Seeds of *Solanum lycopersicum* cv IPA-5 and *Arachis pusilla* were kindly supplied by Instituto Agronômico de Pernambuco (IPA) and Prof. Reginaldo de Carvalho (Universidade Federal Rural de Pernambuco), respectively. The remaining species were weeds or cultivated plants growing on the campus of the Federal University of Pernambuco, Recife, Brazil. A list of all investigated species is presented in Table 1.

**Table 1:** List of species investigated with the karyological data observed here. The species were ordered from the lowest to the highest genome size.

Species	(2n)	CP	H4K5ac	2C (pg) ± SD	Standard	2C/2n (pg)
***Bixa orellana* L.**	14	S	L	0.66 ± 0,03	*Glycine max* Merr “Polanka”	0.05
***Poncirus trifoliata* L. (Raf)**	18	S	L	0.78^a^	-	0.04
***Rhynchospora tenuis* Link**	4	Al	U	0.78 ± 0.05	*Solanum lycopersicum* L. “Stupické	0.19
***Eleocharis geniculata* (L.) Roem. & Schult.**	10	Al	U	1.14 ± 0.01	*S*. *lycopersicum*	0.11
***Phaseolus vulgaris* L.**	22	S	L	1.20^a^	-	0.05
***Phaseolus lunatus* L.**	22	S	L	1.40^a^	-	0.06
***Emilia sonchifolia* (L.) DC**	10	H	G	1.44 ± 0.06	*G*. *max*	0.14
***Indigofera campestris* Bong. ex Benth.**	32	S	L	1.85 ± 0.18	*Zea mays* L. “CE-333”	0.06
***Solanun lycopersicum* L. cv. IPA-5**	24	S	L	2.13 ±0.04	*Z*. *mays*	0.08
***Eleutherine bulbosa* (Miller) Urban**	12	S	L	2.63 ± 0.12	*Z*. *mays*	0.22
***Rhynchospora pubera* L.**	10	Al	U	3.29 ± 0.18	*Z*. *mays*	0.33
***Costus spiralis* (Jacq.) Roscoe**	18	S	L	3.32 ± 0.12	*Pisum sativum* L. “Ctirad”	0.18
***Arachis pusilla* Benth**	20	S	L	4.18 ^a^	*-*	0.21
***Callisia repens* (Jacq.) L.**	12	Al	U	16.53 ± 0.24	*Vicia faba* L. “Inovec”	1.38
***Allium cepa* L.**	16	Al	U	33.55^a^	-	2.09
***Araucaria angustifolia* (Bertol.) Kuntze**	26	Al	U	44.7 ^a^	-	1.72
***Nothoscordum pulchellum* Kunth.**	10	Al	U	49.94 ± 0.33^b^	*V*. *faba*	4.99

^a^http://data.kew.org/cvalues/

^b^(Gustavo Souza, personal communication)

Abbreviations: 2n (chromosome number); CP (condensation pattern); H4K5ac (distribution of anti-H4K5ac signals); 2C (nuclear DNA content); Standard (standard species used as reference for DNA content estimation); 2C/2n (average DNA content per chromosome); Ar (Arabidopsis-type); Al (Allium-type); H (Hordeum-type); S (Solanum-type); U (uniform); L (localized); G (gradual).

For Giemsa and CMA/DAPI staining, root tips were pretreated in 0.002 M 8-hydroxyquinoline (for species with small to medium-sized chromosomes) or 0.2% colchicine (for large chromosomes) for 24 hours at 10°C, fixed in a 3:1 ethanol-acetic acid solution, and subsequently stored at −20°C. For immunostaining, the roots tips were pretreated as above, fixed for 40 min in 4% paraformaldehyde, and subsequently rinsed in PBS.

### Giemsa and CMA/DAPI staining and H4K5ac immunodetection

The protocols used for Giemsa and fluorochrome staining were identical to those previously used with most of the species examined here (see *e*.*g*., [12]). Briefly, for conventional staining with Giemsa, young root tips were washed in distilled water, hydrolyzed in 5N HCl, and squashed in 45% acetic acid. The coverslips were removed in liquid nitrogen and the slides stained with 2% Giemsa. For CMA/DAPI double staining, the root tips were digested in a 2% cellulase-20% pectinase solution and squashed in 45% acetic acid. The coverslips were removed in liquid nitrogen and the slides were left to age for three days. They were then stained with CMA (0.1 mg/ml) for 60 min and subsequently counterstained with DAPI (2 μg/ml). The slides were then mounted in glycerol/McIlvaine (1:1) and kept in the dark for three days before analysis.

The protocol for immunostaining was identical to that used with *Costus* and *Eleutherine* by Feitoza and Guerra [21]. The root tips were digested in a cellulase-pectinase solution and squashed in PBS buffer. The coverslips were removed in liquid nitrogen and the slides incubated in a blocking solution with 3% BSA (w/v) containing 0.1% Triton X-100 in PBS. The primary anti-H4K5ac antibody used (rabbit polyclonal IgG, Upstate Biotechnology, USA) was diluted 1:300 in PBS containing 3% BSA. The slides were incubated overnight at 4°C and detected with FITC-conjugated anti-rabbit IgG (Sigma) diluted 1:60 in a blocking solution. After washes in 1× PBS, the preparations were mounted and counterstained with DAPI (2 μg/mL):Vectashield (1:1). Photographic records were made using a Leica DMLB microscope equipped with a CCD Cohu video camera, and Leica QFISH software. Final images were edited using Adobe Photoshop CS3 version 10.0 software.

### Nuclear DNA contents

DNA content estimations were performed using a CyFlow SL (Partec) flow cytometer and Flomax software (Partec), following the protocol described by Doležel *et al*. [38]. Young leaves of the specimens examined were fragmented (using a razor blade) together with an internal standard, in 0.5 ml of cold Otto I buffer (0.1 M citric acid + 0.5% Tween 20), and filtered through a nylon membrane (45 μm pores). After the addition of 0.5 ml Otto II buffer (0.4 M Na_2_HPO_4_.12H_2_O) supplemented with RNAse (50 μg/ml) and propidium iodide (PI) (50 μg/ml), the samples were immediately analyzed. Exceptionally, for *Callisia repens*, fragmentation was performed in WPB (woody plant buffer), followed by filtering, and supplemented with RNAse and PI, following Loureiro *et al*.[39].

The internal reference standards used were: *S*. *lycopersicum* L. ‘Stupické polní rané’ (2C = 1.96 pg), *Vicia faba* L. ‘Inovec’ (2C = 26.90 pg), *Pisum sativum* L. ‘Ctirad’ (2C = 9.09 pg), *Zea mays* L. ‘CE-777’ (2C = 5.43 pg), and *Glycine Max* (L.) Merr. ‘Polanka’ (2C = 2.55 pg) [38]. Seeds of all of the internal standards were furnished by Dr. J. Doležel (Institute of Experimental Botany, Olomouc, Czech Republic). An internal standard was selected for each plant analyzed so that its genome size was close to, but not overlapping, that of the analyzed sample. Nuclear DNA contents (2C) were calculated by the equation: (Sample peak mean/Standard peak mean) × 2C DNA content of standard (pg). Each species was evaluated three different times on three different days. We also estimated the genome sizes of *E*. *bulbosa* and *C*. *spiralis* from the same cultivated samples previously analyzed for their H4K5ac distributions [21].

## Results

### Estimates of nuclear DNA content

The DNA contents of *Callisia repens*, *Eleocharis geniculata*, *Emilia sonchifolia*, *Indigofera campestris*, *Rhynchospora pubera*, *R*. *tenuis*, *C*. *spiralis*, and *E*. *bulbosa* were estimated here for the first time and are presented in Table 1, together with the internal standard used for each. The 2C values previously estimated for the remaining species (*A*. *cepa*, *A*. *angustifolia*, *Arachis pusilla*, *Bixa orellana*, *Nothoscordum pulchellum*, *Phaseolus vulgaris*, *P*. *lunatus*, and *Poncirus trifoliata*) and their respective average chromosomal DNA contents (2C/2n values) are also indicated in Table 1. The value obtained for *B*. *orellana* (2C = 0.66 ± 0.03) was ~50% greater than previously reported [40]. In order to check for technical errors, we analyzed a second sample collected in a private garden using a different standard species (*Raphanus sativus* cv. Saxa, 2C = 1.11, [38]) as well as a different buffer (WPB), but the 2C value obtained remained nearly the same (0.65 ± 0.02 pg). The smallest 2C and 2C/2n values observed here were for *B*. *orellana* and *P*. *trifoliata* (0.66 and 0.04 pg respectively) while the highest value found was with *N*. *pulchellum* (2C = 49.94 pg; 2C/2n = 4.99 pg).

### Correlation between CP and DNA content

**Fig 1:**
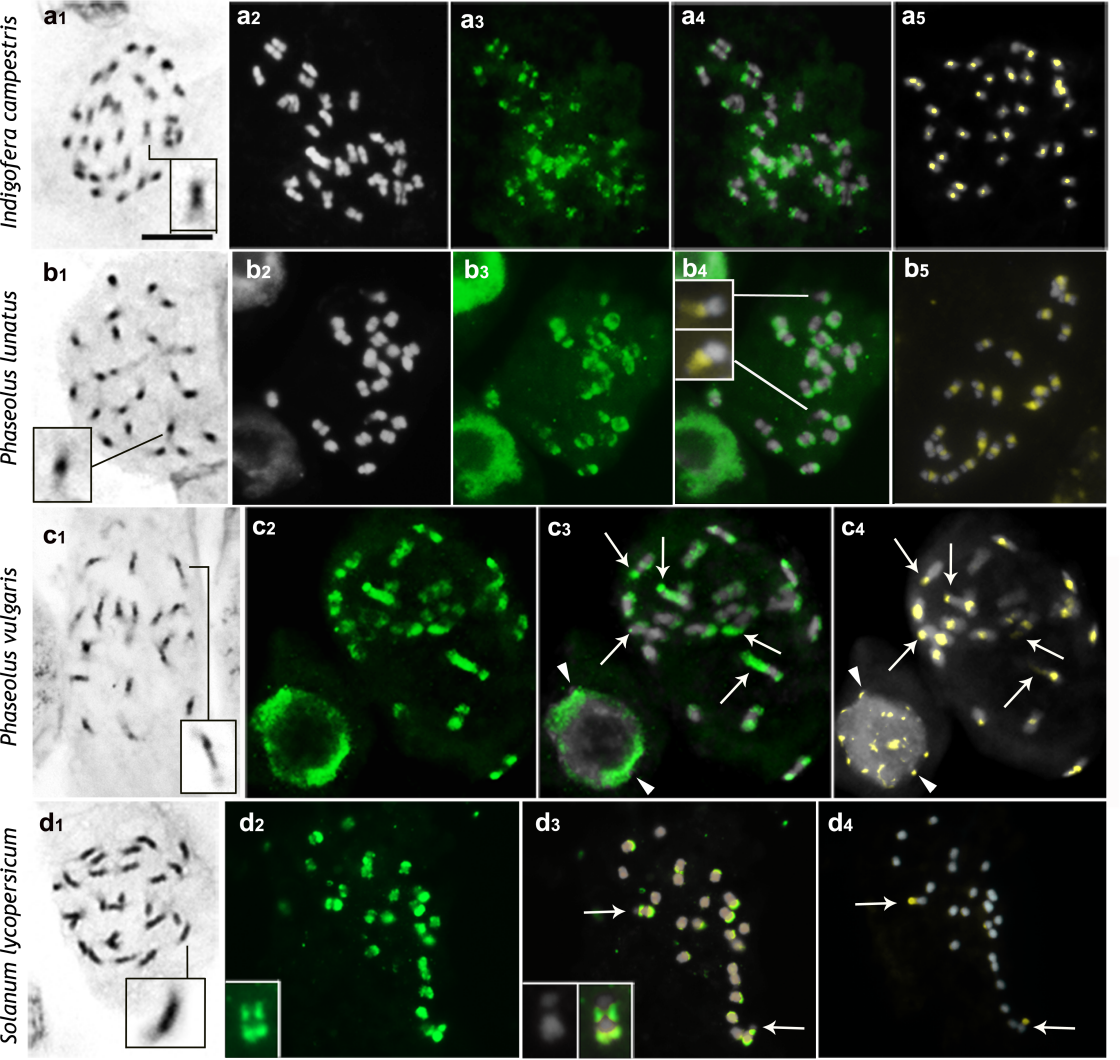
Distribution of H4K5ac in chromosomes of species with low DNA content. a, *Indigofera campestris*; b, *Phaseolus lunatus*; c, *P*. *vulgaris*; d, *Solanum lycopersicum*. Images display, respectively, Giemsa (black and white), DAPI (gray), FITC-conjugated anti-H4K5ac (green), DAPI/FITC (gray/green), and CMA/DAPI (yellow/gray) staining. Insets show magnified chromosomes displaying high and low condensed regions (a1-d1), and CMA^+^ NORs unlabeled with anti-H4K5ac (b4, b5). Arrows in c and d point to hyperacetylated CMA^+^ NORs. Insets in d2 and d3 show magnified chromosome (left arrow). Arrowheads in c3 and c4 indicate hypoacetylated CMA^+^ chromocenters. Scale bar in a1 corresponds to 10 μm.

**Fig 2:**
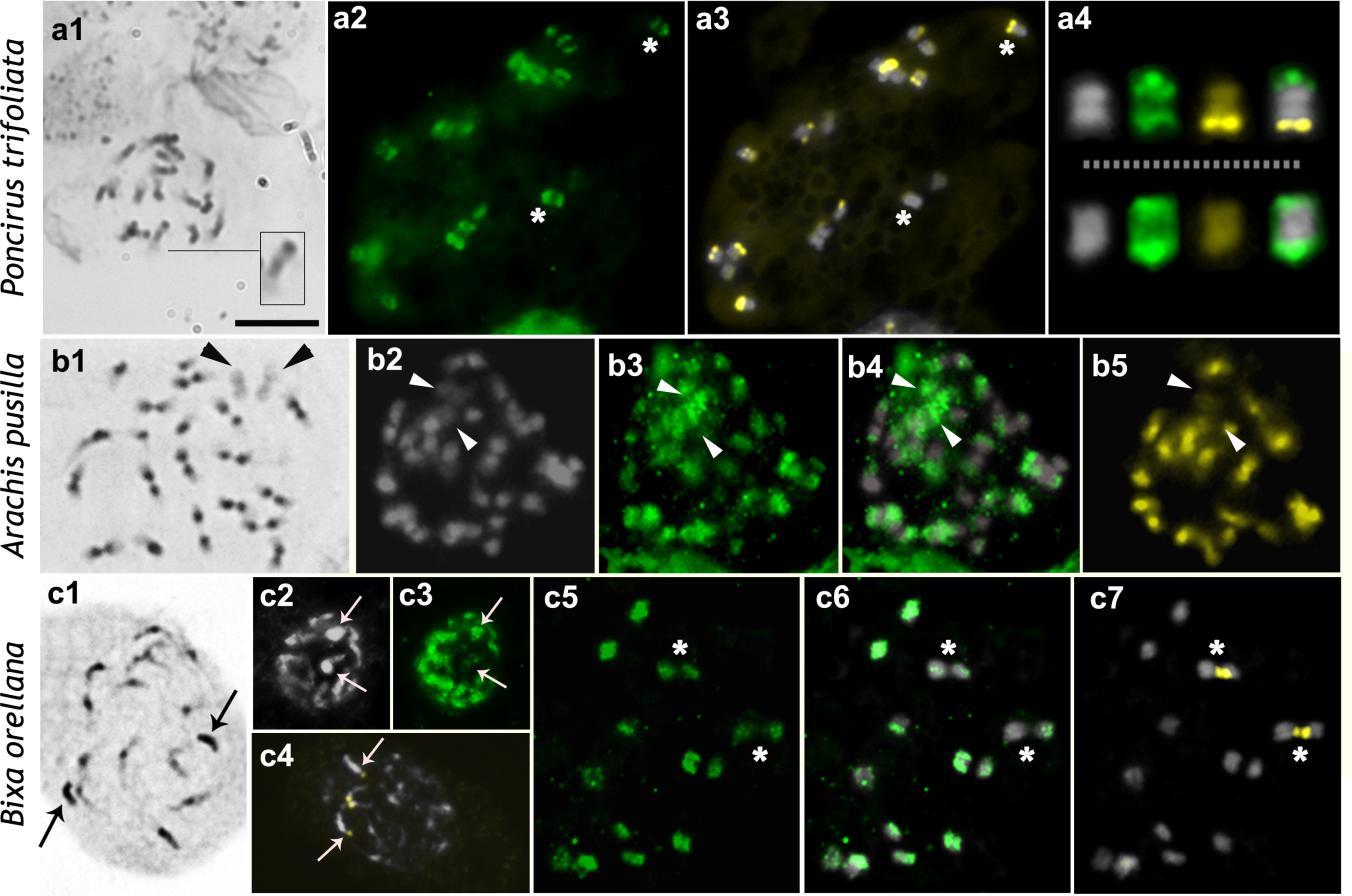
Distribution of H4K5ac in chromosomes of species with low DNA content. a, *Poncirus trifoliata*; b, *Arachis pusilla*; c, *Bixa orellana*. Images display, respectively, Giemsa (black and white), DAPI (gray), FITC-conjugated anti-H4K5ac (green), DAPI/FITC (gray/green), CMA (yellow), and CMA/DAPI (yellow/gray) staining. Inset in a1 shows a chromosome with a proximal early condensing chromatin and a terminal heterochromatic block. Arrowheads in b point to the weakly stained A chromosome pair. Asterisks in a indicate chromosomes amplified in a4. Arrows in c point to the heterochromatic arm of chromosome pair 1 in prophase nuclei. Asterisks in c indicate the hypoacetylated CMA^+^ regions around the secondary constriction. Scale bar in a1 corresponds to 10 μm.

Chromosomes of most species with low DNA content (2C < 5 pg) displayed typically well condensed regions (deeply stained with Giemsa), and less condensed regions (weakly stained) (Figs 1 and 2). In species with the Solanum-type CP, the amount of early condensing chromatin was quite variable, depending on the species investigated. A typical Arabidopsis-type CP was not found. The group of species with Solanum-type CP included *B*. *orellana* (2C = 0.66 pg), *P*. *trifoliata* (2C = 0.78 pg), *P*. *vulgaris* (2C = 1.20 pg), *P*. *lunatus* (2C = 1.40 pg), *I*. *campestris* (2C = 1.85 pg), *S*. *lycopersicum* (2C = 2.13 pg), *E*. *bulbosa* (2C = 2.63 pg), *C*. *spiralis* (2C = 3.32 pg), and *A*. *pusilla* (2C = 4.18 pg) (Table 1). *Bixa orellana* stood out for having a bimodal karyotype, with six small chromosome pairs and a larger one. During prometaphase, one of the arms of the largest chromosome pair was more deeply stained than the other (Fig 2C1 and 2C4). This chromosome pair had a proximal secondary constriction that was usually highly distended, leaving the chromosome arms far from each other (arrows in Fig 2C4 point to the heterochromatic arms whereas the other two arms were associated with each other by the CMA^+^ NORs). Likewise, *E*. *bulbosa* had a bimodal karyotype with a single pair of large chromosomes (~8 μm, corresponding to 42.5% of the total chromosome complement length or 0.56 pg) that were almost entirely condensed during prometaphase, except for a very small terminal region, whereas the five pairs of small chromosomes displayed overall low condensation [41].

**Fig 3:**
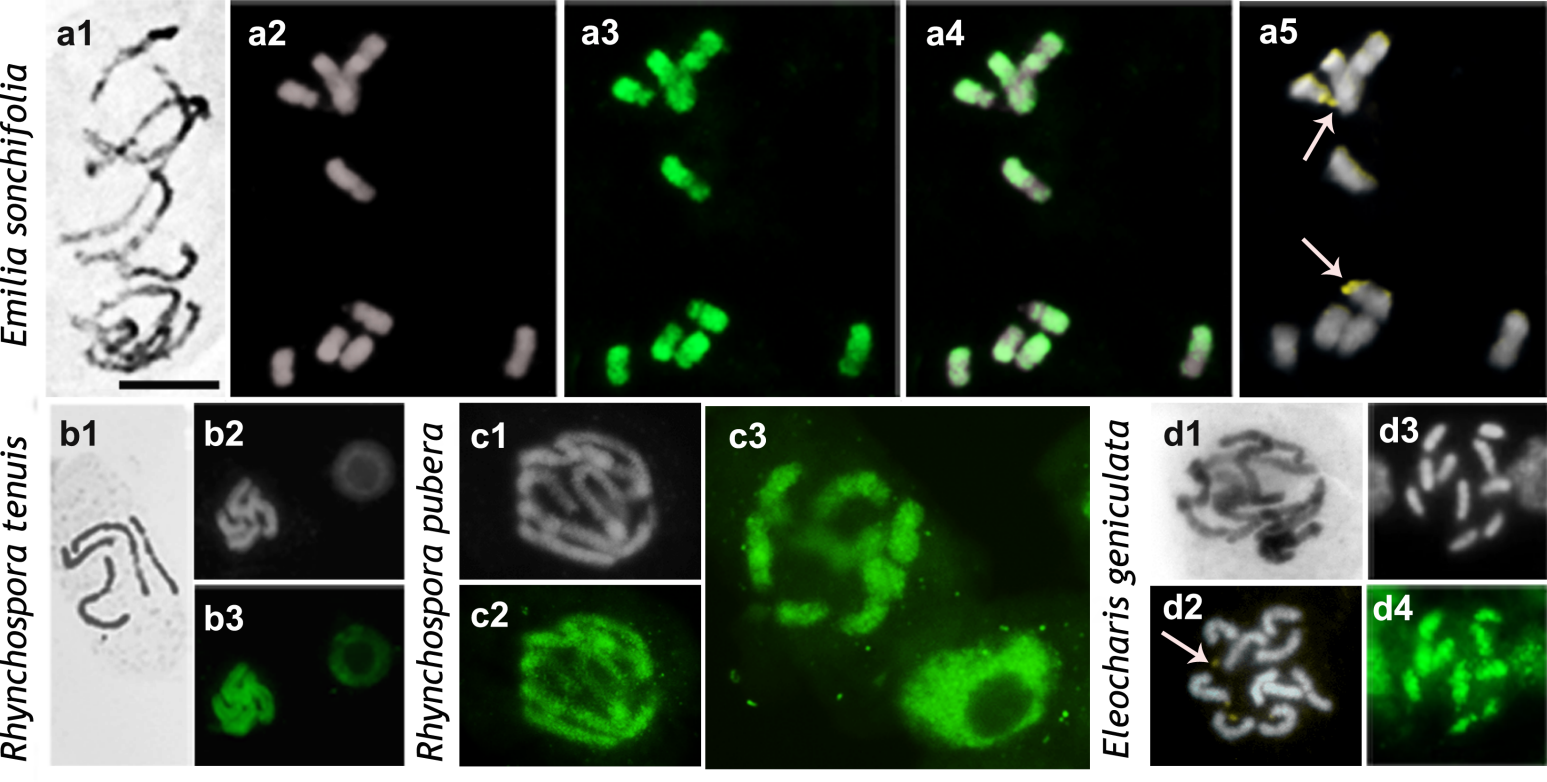
Distribution of H4K5ac in chromosomes of *Emilia sonchifolia* (**a**), *Rhynchospora tenuis* (**b**), *R*. *pubera* (**c**), and *Eleocharis geniculata* (**d**). Images display, respectively, Giemsa (black and white), DAPI (gray), FITC-conjugated anti-H4K5ac (green), DAPI/FITC (gray/green), and CMA/DAPI (yellow/gray) staining. Arrows in a5 and d2 point to NORs. Scale bar in a1 corresponds to 10 μm.

The genome of *E*. *sonchifolia* (2C = 1.44 pg) was slightly larger than that of the other species with low nuclear DNA content (Table 1). Its chromosomes were almost uniformly condensed during prophase and prometaphase, with the short arms being more condensed than the long arms (Fig 3A1) due to their pericentromeric heterochromatin [42], whereas the interstitial to terminal regions of the long arms were slightly less condensed (Hordeum-type CP).

**Fig 4:**
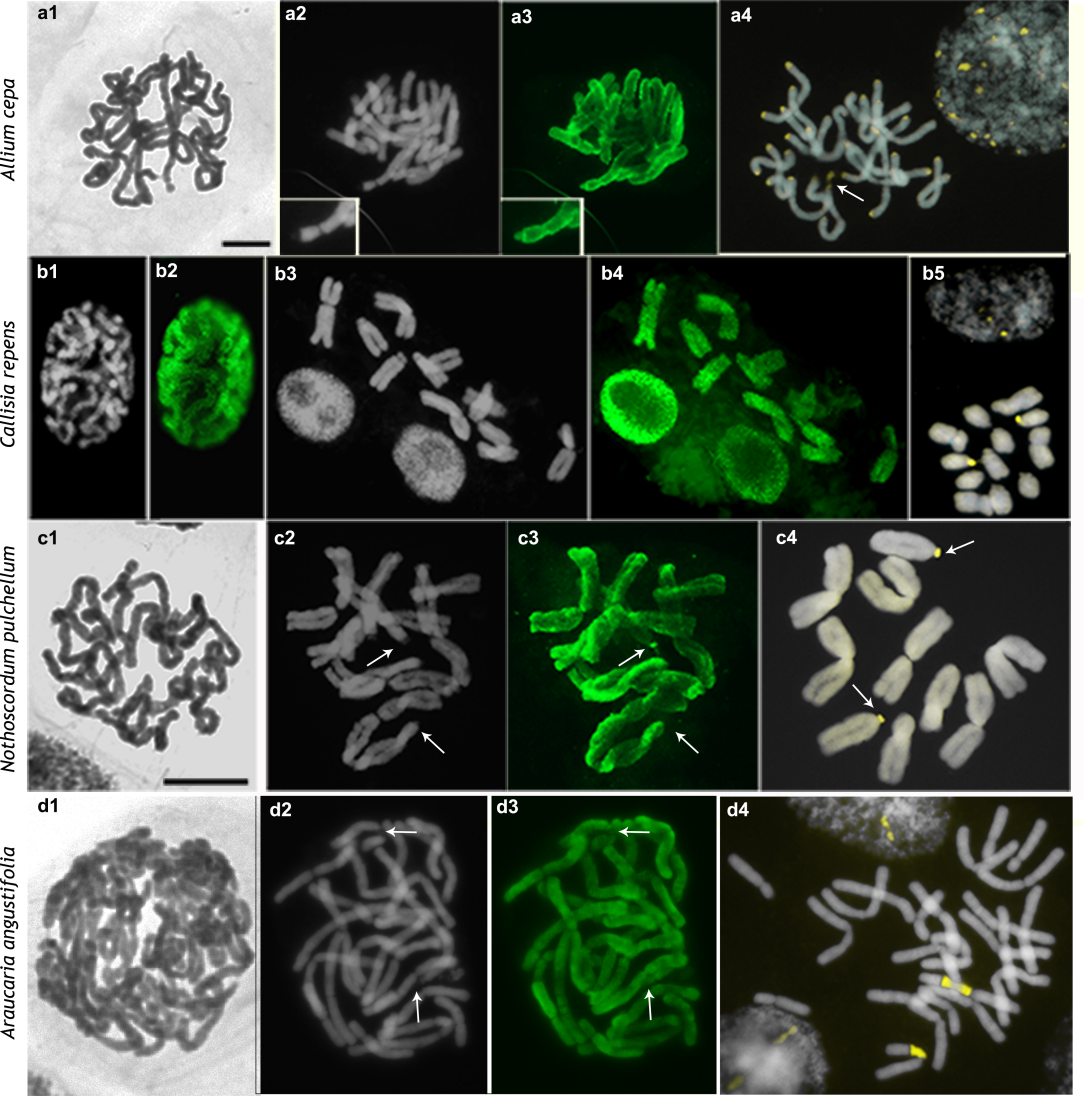
Distribution of H4K5ac in chromosomes of species with high DNA content. a, *Allium cepa*; b, *Callisia repens;* c, *Nothoscordum pulchellum*; d, *Araucaria angustifolia*. Images display, respectively, Giemsa (black and white), DAPI (gray), FITC-conjugated anti-H4K5ac (green), and CMA/DAPI (yellow/gray) staining. Arrows point to the NORs. Scale bar in a1 corresponds to 10 μm.

The three other species with low genomic DNA content (*Rhynchospora tenuis*, 2C = 0.78 pg; *R*. *pubera*, 2C = 3.29 pg; *Eleocharis geniculata*, 2C = 1.14 pg) had small to medium sized chromosomes, which were uniformly condensed during prophase (Allium-type CP, Fig 3B1–3D1). The four species with large genomes and large chromosomes (*A*. *cepa*, 2C = 33.55 pg; *C*. *repens*, 2C = 16.53 pg; *N*. *pulchellum*, 2C = 49.94 pg; and *A*. *angustifolia*, 2C = 44.7) also had uniformly condensed prophase chromatin but much denser than the three former species (Fig 4A1, 4C1 and 4D1).

### Heterochromatin differentiation with CMA/DAPI

Staining with the fluorochromes CMA and DAPI identified CMA^+^ bands in one or more chromosome pairs in all of the species investigated. These bands corresponded to all of (or most of) the known C-bands for those species [12,34,36, 39,43–44]. No DAPI^+^ band was clearly observed. Noteworthy, the heterochromatic bands in all species with Solanum-type CP constituted only a small fraction of the condensed chromatin.

The CMA^+^ bands of *I*. *campestris*, *P*. *lunatus*, *P*. *vulgaris*, and *A*. *pusilla* were located in the proximal regions of all of the chromosomes (Figs 1 and 2). In *B*. *orellana* and *A*. *angustifolia* there was a single pair of CMA^+^/DAPI^˗^ band, which was located in the proximal region and corresponded to the NOR (Figs 2C7 and 4D4). Additional pericentromeric heterochromatin on the smaller chromosomes of *B*. *orellana* and interstitial to terminal bands observed on its largest chromosome pair were identified only by C-banding [36] but not by CMA/DAPI staining. In both *Phaseolus* species, besides the proximal CMA^+^ bands, there were additional terminal CMA^+^ bands corresponding to the NORs (Fig 1B5 and 1C4). NORs were identified as secondary constrictions distended at metaphase or the only CMA^+^ bands in the karyotype, as in *B*. *orellana* (Fig 2C7), *E*. *sonchifolia* (Fig 3A5), *N*. *pulchellum* (Fig 4C4), and *A*. *angustifolia* (Fig 4D4). In *P*. *lunatus*, *N*. *pulchellum*, *P*. *trifoliata*, *E*. *sonchifolia*, and *S*. *lycopersicum* the NORs had also been previously identified by FISH with rDNA probe [12,33,35,39,43]. In *S*. *lycopersicum*, besides the NORs there were some very small bands on other chromosomes (Fig 1D4). Small CMA^+^ bands were observed in the terminal regions of all of the chromosomes of *A*. *cepa*, including the NORs localized terminally in the short arms of a submetacentric pair (Fig 4A4). In *P*. *trifoliata* there were 16 CMA^+^ bands, six of which correspond to rDNA sites [33] (Fig 2A3).

### Chromosomal distributions of H4K5ac

The immunodetection of H4K5ac revealed three different chromosome labeling patterns: (a) uniform (chromosomes uniformly labeled), (b) gradual (labeling decreasing in intensity from the terminal region to the proximal one); (c) localized (signals restricted to the low condensed regions). The localized pattern was found in all species with the Solanum-type CP (Table 1; Figs 1 and 2). In *P*. *trifoliata*, the chromosomes with a terminal CMA^+^ band displayed often a subterminal labeling (Fig 2A4, upper row). The small chromosomes of *B*. *orellana* differed from the largest pair in that the former were hyperacetylated in the both interstitial and terminal regions, while the large chromosome pair was weakly and irregularly labeled in these regions (Fig 2C5-7).

Uniformly acetylated prophase/prometaphase chromosomes were found in all species with large genomes (*A*. *cepa*, *C*. *repens*, *N*. *pulchellum*, and *A*. *angustifolia)* or with holokinetic chromosomes (*R*. *tenuis*, *R*. *pubera*, and *E*. *geniculata*) (compare Fig 3B–3D with Fig 4). Labeling had often a granular appearance, sometimes displaying small gaps, quite different from the compact and more brilliant blocks observed in species with differentially condensed chromatin. A gradual labeling pattern was observed only in *E*. *sonchifolia*, which displayed chromosomes more acetylated in the terminal region of the long arms and less acetylated in the short arms and pericentromeric regions (Fig 3A3-4). Remarkably, NORs were the only chromosomal regions that could be unequivocally identified as non-acetylated (Figs 1B4, 2C5-7 and 3A3-5), hypoacetylated (Fig 4A3, 4C3 and 4D3) or hyperacetylated (Fig 1C3 and 1D3), depending on the species.

In prophase to metaphase cells of *A*. *pusilla* and most other species of the section *Arachis*, there is a small, typically under-condensed chromosome pair, called A chromosome [45]. Even at metaphase this kind of chromosome displayed only a very small amount of completely condensed chromatin and was entirely hyperacetylated (Fig 2B2-5). Observe that the A chromosomes were very weakly stained with both DAPI (Fig 2B2) and CMA (Fig 2B5).

## Discussion

### Chromosome condensation pattern and DNA content

All of the species with the Solanum-type CP that were investigated here, including *E*. *bulbosa* and *C*. *spiralis*, had low nuclear DNA content (2C < 5 pg) and low mean DNA content per chromosome (2C/2n < 0.5 pg). The species with the Allium-type CP had either large genomes (2C > 5 pg) and large amount of DNA per chromosome (> 0.5 pg), or small genomes and holokinetic chromosomes. Holokinetic chromosomes are characterized by multiple centromeric units intercalated with small patches of euchromatin [46]. This regular and punctate distribution of euchromatin could explain the uniform acetylation pattern found in these species, independent of genome size. A small genome size was also found in *Emilia sonchifolia*, the only species in the present sample displaying the Hordeum-type CP. Therefore, the amount of DNA per genome (or per chromosome) is associated with the different CPs but it is not enough to determine them. A similar correlation between nuclear/chromosomal DNA content and CP had been previously observed in 17 species of Rutaceae [17]. Likewise, species with areticulate and semi-reticulate interphase nuclei (associated with Arabidopsis/Solanum-type CP) had low DNA content (< 5 pg) whereas those with reticulate and eureticulate nuclei (Hordeum/Allium-type) had higher DNA content [16]. In both these studies there were some exceptions to the general trend, suggesting that other factors may also contribute to the establishment of a specific CP.

### Chromosome condensation pattern and H4K5 acetylation

The H4K5 acetylation patterns observed here are in agreement with those reported for other plant species with known CPs, such as *A*. *thaliana*, *Silene latifolia*, *V*. *faba*, and *H*. *vulgare* [9,20,22]. Our data further confirmed the acetylation pattern previously described for *A*. *cepa* [22] and *Phaseolus vulgaris* [28], reinforcing the reliability of the methodology used.

In the present sample we found three different patterns of H4K5ac distribution: an uniform labeling, in species with the Allium-type CP; a gradual labeling, associated with the Hordeum-type CP; and a localized labeling, in species with the Arabidopsis/Solanum-type CP. These patterns were quite similar to those observed for H3K4me2 [18,47], suggesting that both histone modifications contribute to determine the chromatin condensation timing and CPs.

Notably, all of the chromosomes of a karyotype display the same CP and similar distribution of H4K5ac [19,22,28], H3K4me2 [18,47], and some other epigenetic marks [21,30,47]. However, the proportion of high/low condensed chromatin may vary between prophase/prometaphase chromosomes of a karyotype exhibiting the Arabidopsis/Solanum-type PC. For example, during the prometaphase of *E*. *bulbosa* only the chromosome pair I was almost entirely condensed and unlabeled with anti-H4K5ac [21], whereas in *A*. *pusilla* only the A chromosome pair was almost completely undercondensed. The differential proportion of high and low condensed chromatin observed in these chromosome pairs was directly correlated with the amount of repetitive DNA sequences accumulated in each one. In *E*. *bulbosa*, the main satellite DNA sequences and most retroelements are largely accumulated in the chromosome pair I [48], while in *Arachis* species the A chromosome pair exhibit the lowest concentration of repetitive sequences [49]. Likewise, the more condensed chromatin fraction of prophase/prometaphase chromosomes of *S*. *lycopersicum* is highly enriched in retrotransposons and has a gene density 10–100 times lower than the less condensed euchromatin [50, 51]. Therefore, the CP should be primarily determined not by chromosome size or chromosomal DNA content but rather by the amount of repetitive sequences and by the way they are distributed along the chromosomes.

### H4K5 acetylation levels in euchromatin and heterochromatin

In prophase/prometaphase chromosomes of species displaying the Arabidopsis/Solanum-type CP, the less condensed euchromatin fraction differed strongly from the more condensed one in their acetylation levels, indicating that hyperacetylation is not a universal mark of euchromatin (at least when we consider the euchromatin concept based on C-banding). For example, the interstitial to proximal condensed region of most prophase/prometaphase chromosomes of *S*. *lycopersicum* includes only a small fraction of heterochromatin [12] but it is uniformly non-acetylated.

Heterochromatic bands, on the other hand, are known to be cytologically lacking of H4K5ac signals [18,21] and indeed most of them were non-acetylated. However, the pericentromeric C-bands present in all of the chromosomes of *E*. *sonchifolia* [42] and the terminal heterochromatic bands of *A*. *cepa*, as well as the pericentromeric C-bands of *A*. *cepa* reported by some authors [32,52], were weakly acetylated (see also Vyskot et al. [22]). The largest chromosome pair of *B*. *orellana* had one chromosome arm completely heteropycnotic, mainly composed by C-bands [34], which was partially labeled with anti-H4K5ac. These data indicate that some heterochromatic regions may be considerably enriched in H4K5ac, as previously observed in other organisms [24,29,53–54].

A particular kind of heterochromatin positively stained for C-banding and CMA is the NOR [42]. In the present sample, NORs could be hyperacetylated (*Phaseolus vulgaris*, *P*. *lunatus*, and *S*. *lycopersicum*), weekly acetylated (*A*. *cepa*, *A*. *angustifolia*, and *N*. *pulchellum*), or non-acetylated (*B*. *orellana*, *E*. *bulbosa*, *P*. *trifoliata*, and *E*. *sonchifolia*). The acetylation level in these cases was independent of the condensation status of the NORs in prophase and metaphase cells. This is an unusual condition where a CMA^+^ band (NOR) can be hyperacetylated [19–21,23] and the acetylation pattern does not match perfectly the heteropycnotic pattern.

## Conclusions

Our data indicate that chromosome condensation is a karyotype feature that affects all chromosomes of a complement in a similar way. This is most evident in chromosomes with Allium-type CP, but it is equally true for all other CPs. The different patterns are strongly associated with the chromosomal distribution of H4K5ac and only partially correlated with nuclear and mean chromosomal DNA contents. The repetitive DNA sequences of species with the Arabidopsis/Solanum-type CP are largely concentrated in the more condensed and non-acetylated proximal chromosome regions, whereas in Allium/Hordeum-type CP they are apparently finely spread throughout of the chromosomes. The change from one distribution pattern to the other is in some way related to changes in the average DNA amount per chromosome, but other karyological features, as for example massive expansion of repetitive sequences or the presence of holocentromeres, may play a dominant role in the establishment of a new CP in all chromosomes of the complement.
